# Impact of serum interleukin-22 as a biomarker for the differential use of molecular targeted drugs in psoriatic arthritis: a retrospective study

**DOI:** 10.1186/s13075-022-02771-4

**Published:** 2022-04-15

**Authors:** Ippei Miyagawa, Shingo Nakayamada, Masanobu Ueno, Yusuke Miyazaki, Shigeru Iwata, Satoshi Kubo, Koshiro Sonomoto, Junpei Anan, Naoaki Ohkubo, Yoshino Inoue, Yoshiya Tanaka

**Affiliations:** 1 The First Department of Internal Medicine, University of Occupational and Environmental Health, Japan, 1-1 Iseigaoka, Yahata-nishi, 807-8555 Kitakyushu, Japan; 2 Mitsubishi Tanabe Pharma Corp, Yokohama, Japan

**Keywords:** Psoriatic arthritis, Precision medicine, Interleukin-22, Tumor necrosis factor inhibitors, Interleukin-17 inhibitors

## Abstract

**Background:**

We explored whether serum cytokines could be used as biomarkers for optimal use of tumor necrosis factor inhibitors (TNF-i) and interleukin (IL)-17 inhibitors (IL-17-i) in patients with psoriatic arthritis (PsA).

**Methods:**

In cohort 1 (47 patients treated with IL-17-i [*n*=23] or TNF-i [*n*=24] for ≥1 year), we identified serum cytokines that predicted the achievement of Disease Activity in Psoriatic Arthritis-remission (DAPSA-REM), Psoriasis Area and Severity Index (PASI) 90, and Minimal Disease Activity after 1 year of TNF-i or IL-17-i therapy. Subsequently, we developed treatment strategies based on the identified cytokines; initiation of IL-17-i therapy in patients with low IL-22 concentrations (IL-22 <0.61376 pg/ml) and TNF-i therapy in patients with high IL-22 concentrations (0.61376< IL-22 pg/ml). In cohort 2 (34 patients), treatment responses were compared between the strategic treatment group (*n*=17), which was treated based on the treatment strategies, and the mismatched treatment group (*n*=17) to verify the validity of the treatment strategies developed using serum cytokines as biomarkers.

**Results:**

In cohort 1, serum IL-22 concentration was identified as a predictor of DAPSA-remission after 1 year of IL-17-i therapy. Regarding treatment strategies, we selected TNF-i for patients with high IL-22 concentrations and IL-17-i for those with low IL-22 concentrations. There were no significant differences in the baseline characteristics between the strategic and mismatched treatment groups. Regarding treatment effects, activity significantly improved at 1 year in both groups. Upon comparison of the treatment effects, the rate of achieving DAPSA-REM and Minimal Disease Activity at month 12 was significantly higher in the strategic treatment group.

**Conclusions:**

The results of this pilot study suggest that IL-22 may be a biomarker of treatment response to TNF-i and IL-17-i in patients with PsA. Further large-scale studies in independent, prospectively collected datasets are required to verify that IL-22 is indeed a biomarker of treatment response in these patients.

**Supplementary Information:**

The online version contains supplementary material available at 10.1186/s13075-022-02771-4.

## Background

Psoriatic arthritis (PsA) is a typical complication of psoriasis (PS) that often causes irreversible destruction and dysfunction of the peripheral joints and/or the spine. Various cytokines, such as interferon (IFN)-γ, interleukin (IL)-12, IL-23, IL-17, IL-6, and tumor necrosis factor (TNF)-α, play an important role in the pathogenesis of PS and PsA. In addition, increases in IL-22-producing CD4^+^ T cells and IL-17-producing CD4^+^ T cells in the peripheral blood are characteristic of PS and PsA. Serum IL-22 concentrations have been reported to be characteristically and significantly higher in patients with PsA than in those with PS [1]. IL-22 is a cytokine produced by Th22 cells, a newly discovered Th cell subset that is positive for chemokine receptor (CCR)10 and expresses the aryl hydrocarbon receptor as the master transcription factor. Similar to helper T (Th) 17 cells, Th22 cells express CCR4 and CCR6. In a study using an in vitro experimental system with human naïve T cells, we demonstrated that stimulating T cell receptors with IL-1β, TNF-α, and IL-6 induces CD3^+^CD4^+^CCR4^+^CCR6^+^CCR10^+^ Th22 cells that produce IL-22 [2].

EULAR recommends administering targeted therapies, such as TNF inhibitors (TNF-i), IL-17 inhibitors (IL-17-i), IL-12/23 inhibitors (IL-12/23-i), Janus kinase inhibitors (JAK-i), and phosphodiesterase 4 inhibitors (PDE4-i), in patients with PsA and PS who fail to adequately respond to synthetic DMARDs [3]. However, some issues remain. For example, some patients are resistant to these drugs and require changes in their treatment strategies. Further, some have only partial response, and others have divergent responses. Although drugs target different molecules, no optimal drug selection method has been established. Studies directly comparing TNF-i and IL-17-i have shown that these drugs are equally effective [4, 5]. The establishment of an optimal selection method for these drugs can contribute to better patient outcomes.

Recently, efforts have been made to establish precision medicine that aims to improve patient outcomes in systemic autoimmune diseases with high heterogeneity. To implement precision medicine, stratification of patients and selective use of molecular targeted drugs is recommended [6]. Indeed, we attempted precision medicine based on the stratification of patients by lymphocyte phenotyping in patient peripheral blood and found that the proportion of T follicular helper cells in the peripheral blood of patients with rheumatoid arthritis was an independent predictor of favorable responses to abatacept therapy [7]. In addition, we demonstrated that excessive B cell differentiation is associated with treatment resistance in anti-neutrophil cytoplasmic antibody-associated vasculitis and that rituximab is more effective in patients with circulating B cell abnormalities [8]. Furthermore, we showed that the rate of achieving low disease activity as assessed by the Simplified Disease Activity Index (SDAI) after 6 months of treatment was significantly higher in 46 patients with PsA, in whom biological therapy was strategically initiated based on phenotyping of peripheral blood lymphocytes in PsA compared to 38 patients who were conventionally treated with biological drugs based on the 2011 and 2015 EULAR recommendations [9]. We reported the possibility of stratification of patients by phenotyping peripheral blood lymphocytes and precision medicine based on the selective use of molecular targeted drugs in systemic autoimmune diseases, such as PsA. However, since phenotyping of peripheral blood lymphocytes is complex and feasible at a limited number of institutions, the development of simple methods using biomarkers to stratify patients and simple treatment strategies based on such methods is needed to promote precision medicine in a real-world clinical setting.

## Methods

### Patients and clinical measurement

This retrospective study aimed to identify predictors of treatment responses to biological drugs in 47 patients with PsA treated with TNF-i or IL-17-i for ≥1 year while focusing on the serum cytokine concentrations at treatment initiation. This study included two cohorts. Cohort 1 consisted of 47 patients diagnosed with PsA based on the Classification Criteria for Psoriatic Arthritis at multiple institutions affiliated to our university hospital, the key station (FIRST registry). The patients were treated with TNF-i (24 cases) or IL-17-i (23 cases) for ≥1 year between February 2015—when IL-17-i (secukinumab) was approved for reimbursement under the National Health Insurance system for the first time in Japan—and 2018. Cohort 2 (validation cohort) consisted of patients who started treatment with TNF-i (20 cases) or IL-17-i (14 cases) between 2019 and 2020 and were treated for ≥1 year. All patients in both cohorts had peripheral arthritis. The biological drugs were selected based on shared decision-making between the attending physicians and patients. Disease activity was assessed up to 1 year after initiation of the biological therapies in both cohorts. The treatment effects of the biological drugs were assessed using the Disease Activity in Psoriatic Arthritis (DAPSA) and Psoriasis Area and Severity Index (PASI). In addition, the Minimal Disease Activity was evaluated. Meanwhile, seven patients in cohort 1 and five patients in cohort 2 with PASI at 0 at baseline were excluded from the calculation of PASI response rates (PASI75 and PASI90). In cohort 2, the serum cytokine concentrations of all the patients were measured after ≥1 year treatment. The treatment was discontinued due to adverse events in one patient each in both cohorts. In these patients, the last observation carried forward (LOCF) method was used to impute the missing data with the latest available data.

### Establishment of treatment strategy based on serum cytokine concentration

Treatment strategies were established based on the data on serum cytokine concentrations in cohort 1 (study 1). In the 24 patients treated with TNF-i and the 23 patients treated with IL-17-i, univariate logistic analyses were separately performed with achievement of DAPSA remission (REM), PASI90, and Minimal Disease Activity at 1 year as an objective variable. In addition, each serum cytokine concentration was used as an explanatory variable to identify serum cytokines that predicted the achievement of DAPSA-REM, PASI90, and Minimal Disease Activity at 1 year separately in the TNF-i-treated and IL-17-i-treated groups. Furthermore, we developed treatment strategies based on the IL-22 cut-off value of 0.61376 (sensitivity, 81.8%; specificity, 91.7%; area under the curve, 0.848), which was identified as a predictor for DAPSA-REM after 1 year of IL-17-i therapy. The efficacy of the treatment strategies developed in cohort 1 was validated in cohort 2 (validation cohort) (study 2).

### Validation of the treatment strategy in cohort 2

**Fig. 1:**
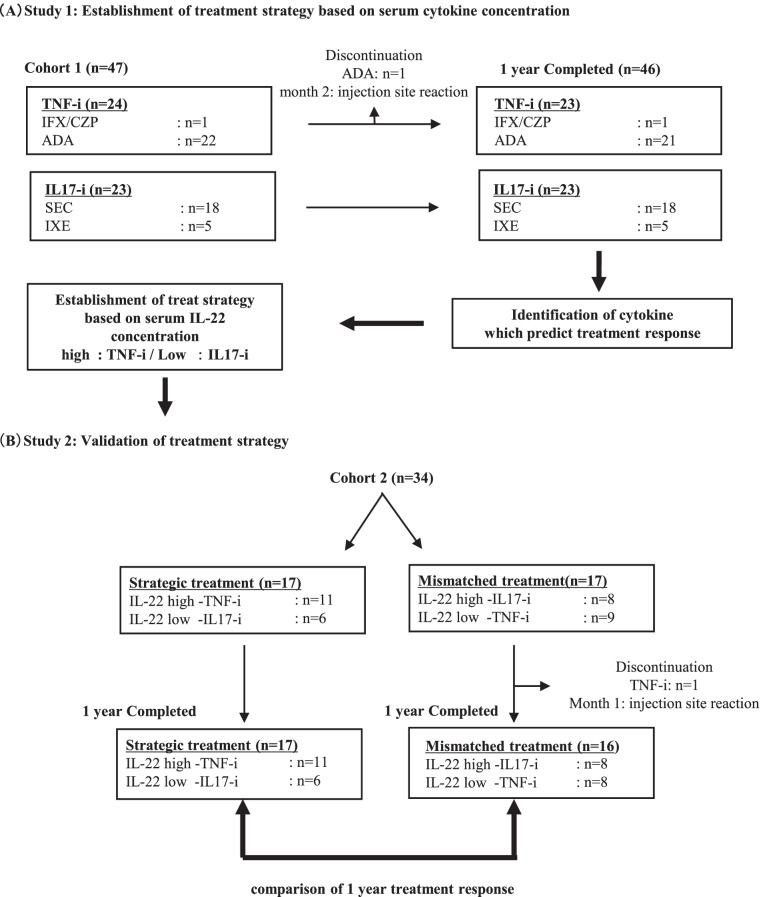
Study design

The treatment effects over 1 year were retrospectively compared between the strategic treatment group, which included 17 patients whose treatment matched the treatment strategies developed based on the results obtained from cohort 1, and the mismatched treatment group, which included 17 patients. Specifically, the strategic treatment group included patients with low IL-22 concentrations who were treated with IL-17-i and those with high IL-22 concentrations who were treated with TNF-i, whereas the mismatched treatment group included patients with low IL-22 concentrations who were treated with TNF-i and those with high IL-22 concentrations who were treated with IL-17-i. The efficacy of the biological drugs over 1 year was compared between these groups to assess the validity of the treatment strategies (Fig. 1B).

### Serum cytokine measurement

In cohorts 1 and 2, serum cytokine (IFN-γ, IL-6, TNF-α, IL-17A, IL-21, IL-22, and IL-23) concentrations were measured. Similarly, these concentrations were also measured in the healthy control (HC) group, matched for age and sex. The concentrations of cytokines at the initiation of bDMARD therapy were measured after a 1-year observation period in all patients in both cohorts. The MESO SCALE DISCOVERY S-PLEX Human IL-17A kit (#K15067L-1, Meso Scale Diagnostics, LLC, Rockville, MD, USA) was used to measure IL-17A (fg/ml), and the U-PLEX Biomarker Group 1 (#K151C3S-1, Meso Scale Diagnostics, LLC, Rockville, MD, USA) was used to measure IFN-γ (pg/ml), IL-6 (pg/ml), TNF-α (pg/ml), IL-21 (pg/ml), IL-22 (pg/ml), and IL-23 (pg/ml) [10].

### Statistical analysis

Data are expressed as mean ± standard deviation, median (interquartile range [IQR]), or number (%). Differences between groups were compared using the Mann-Whitney U test, Kruskal-Wallis test with Dunn’s correction for multiple comparisons or the chi-square test. The Wilcoxon signed rank test was used to identify statistically significant differences between the baseline data and those measured at months 6 and 12. In cohort 1, univariate logistic analyses were performed using the achievement of DAPSA-REM, PASI90, and Minimal Disease Activity at 1 year as an objective variable and serum concentrations of IFN-γ, IL-6, TNF-α, IL-17A, IL-21, IL-22, and IL-23 as explanatory variables to identify serum cytokines that predicted achievement of DAPSA-REM, PASI90, and Minimal Disease Activity at 1 year separately in the TNF-i-treated and IL-17-i-treated groups. All reported *P*-values were two-sided. The level of significance was set at *p*<0.05. All analyses were conducted using JMP Pro version 15 (SAS Institute Inc., Cary, NC, USA) and GraphPad Prism 9 (GraphPad Software, San Diego, CA, USA).

## Results

### Identification of serum cytokines that predict bDMARDs treatment response

Supplemental Table S1 shows the baseline characteristics and serum cytokine concentrations at the initiation of the bDMARD therapies in cohort 1 (TNF-i: 24 cases, IL-17-i: 23 cases). In this non-randomized retrospective study, statistically significant differences were found in the prevalence of complications (diabetes mellitus) and serum IL-17A (Supplemental Table S1); meanwhile, disease activity did not differ between the TNF-i-treated and IL-17-i-treated groups. One year after the initiation of bDMARD therapy, disease activity was significantly improved. DAPSA-REM was achieved in 24 patients (51.3%), and PASI90 was achieved in 26 of 40 patients (65.0%). Thirty-three patients (70.2%) achieved Minimal Disease Activity. In addition, there was no difference in efficacy between TNF-i therapy and IL-17-i therapy at 1 year (Supplementary Table S2).

**Fig. 2:**
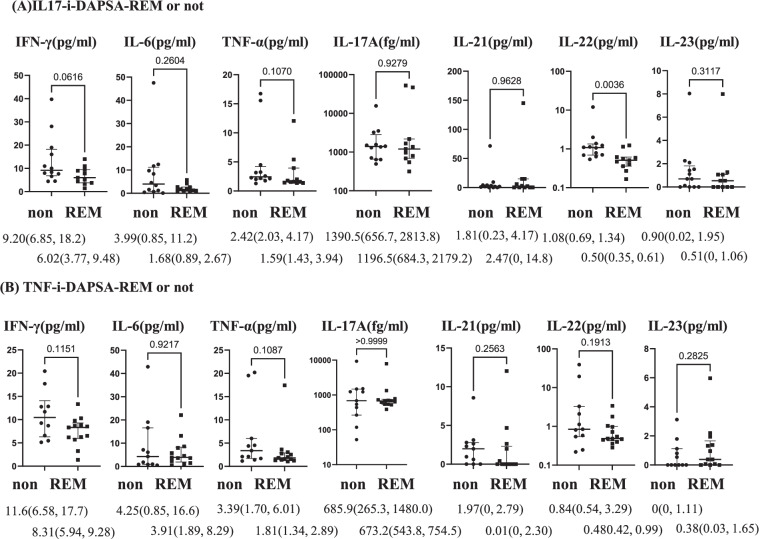
Baseline serum cytokine concentrations in patients who achieved DAPSA-REM and not. Data are presented as median (IQR). **A** Differences in serum cytokine concentrations in patients treated with IL-17-i between patients with DAPSA-REM (*n*=11) and those without (non) (*n*=12). **B** Comparison of serum cytokine concentrations in patients treated with TNF-i between patients with DAPSA-REM (*n*=12) and those without (non) (*n*=12). *p <0.05, Mann-Whitney *U* test

Then, in the TNF-i-treated group (24 cases) and IL-17-i-treated group (23 cases), we performed univariate logistic analyses with the achievement of DAPSA-REM, PASI90, and Minimal Disease Activity as objective variables and serum cytokine concentrations as explanatory variables. Serum IL-22 concentrations were identified as a factor contributing to the achievement of DAPSA-REM in the IL-17-i-treated group. However, no baseline serum cytokines were identified as factors contributing to the achievement of DAPSA-REM in the TNF-i-treated group or achievement of PASI90 and Minimal Disease Activity in either group (Supplementary Tables S3). We compared baseline serum cytokine concentrations among patients who achieved DAPSA-REM (12 cases), those who did not (12 cases), in the TNF-i-treated group and corresponding patients in the IL-17-i-treated group (11 and 12 cases, respectively). The results showed that the baseline serum IL-22 concentrations were significantly lower in patients who achieved DAPSA-REM than in those who did not achieve DAPSA-REM in the IL-17-i-treated group (Fig. 2).

### Establishment of treatment strategy based on serum cytokine concentration

Using a cut-off value of IL-22 0.61376 (sensitivity, 81.8%; specificity, 91.7%; area under the curve, 0.848) determined by a receiver operating characteristic analysis, we stratified 24 patients in the TNF-i-treated group and 23 patients in the IL-17-i-treated group into the IL-22-high group (0.61376< IL-22) and the IL-22-low group (IL-22 <0.61376). Subsequently, the treatment courses over 1 year were retrospectively compared. No significant differences were observed in treatment responses at 1 year. Alternatively, in the IL-17-i-treated group, although the baseline characteristics showed a significantly higher proportion of patients with concomitant use of methotrexate (MTX) at treatment initiation in the IL-22-low group (Supplementary Tables S4), the rate of achieving DAPSA-REM and Minimal Disease Activity after 1 year of IL-17-i therapy was significantly higher in the IL-22-low group (Table 1). Regarding achievement of PASI75/90, no statistically significant differences were observed between the IL-22 high and IL-22-low groups in either the TNF-i-treated or IL-17-i-treated group.

**Table 1:** Comparison of 1 year treatment response between IL-22-high and IL-22-low group

Variables	IL-22 low	IL-22 high	***p*** value
**TNF-i treated group**	*n*=12	n-12	
**DAPSA-REM**	8(66.7)	5(41.6)	0.4136
**DAPSA-LDA**	9(75.0)	10(83.3)	1.0000
**Minimal Disease Activity**	8(66.7)	9(75.0)	1.0000
**PASI 75**	7/11(63.6)	5/11(45.4)	0.6699
**PASI 90**	9/11(81.8)	6 /11(54.5)	0.3615
**IL-17-i-treated group**	*n*=10	*n*=13	
**DAPSA-REM**	9(90)	2(15.3)	*0.0006*
**DAPSA-LDA**	10(100)	12(92.3 %)	1.0000
**Minimal Disease Activity**	10(100)	6(46.1)	*0.0075*
**PASI 75**	7/9(77.8)	7/9(77.8)	1.0000
**PASI 90**	7/9(77.8)	7/9(77.8)	1.0000

Data are expressed as number (%)

*TNF-i* TNF inhibitors, *IL-17-i* IL-17 inhibitors, *DAPSA* disease activity in psoriatic arthritis, *LDA* low disease activity, *REM* remission, *PASI* Psoriasis Area and Severity Index

*p<0.05, by chi-square test

**Fig. 3:**
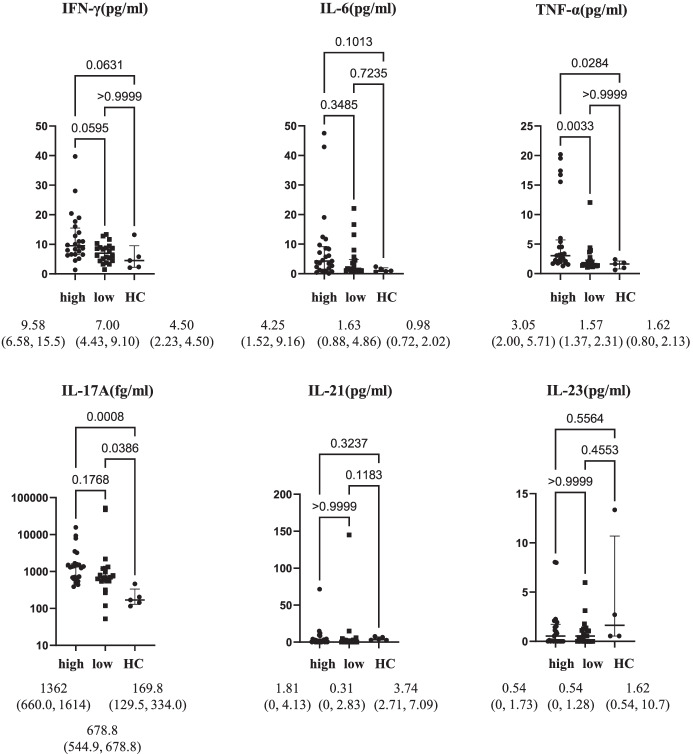
Comparison of cytokine concentration among the IL-22-high group, IL-22-low group, and healthy controls. Data are presented as median (IQR). Serum cytokine concentrations were compared among patients with high IL-22 concentrations (*n*=25), patients with low IL-22 concentrations (*n*=22), and healthy controls (HC) (*n*=5). *p <0.05, by Kruskal-Wallis test with Dunn’s correction

In addition, serum cytokine concentrations were compared among the IL-22 high (25 cases), IL-22 low (22 cases), and HC groups. Serum IL-17 concentrations were significantly higher in both the IL-22 high and IL-22-low groups than in the HC group, whereas no significant difference was observed between the IL-22-high and IL-22-low groups. Alternatively, the serum TNF-α concentrations did not significantly differ between the IL-22 low and HC groups; however, they were significantly higher in the IL-22-high group than in the HC and IL-22-low groups (Fig. 3). Therefore, we assumed that the IL-22-low group was characterized by a preferential increase in serum levels of IL-17 and selected IL-17-i for the IL-22-low group. Conversely, we assumed that the IL-22-high group was characterized by an increase in TNF-α, which can induce differentiation of Th22 cells that produce IL-22, and selected TNF-i to target elevated TNF-α in the IL-22-high group (Fig. 1A).

### Validation of the treatment strategy in cohort 2

To validate the efficacy of the treatment strategies using TNF-i and IL-17-i based on serum IL-22 concentrations, we retrospectively compared the efficacy of the biological drugs at 1 year between the following groups in cohort 2. The strategic treatment group (17 cases) included patients with low IL-22 concentrations who were treated with IL-17-i and those with high IL-22 concentrations who were treated with TNF-i. The mismatched treatment group (17 cases) included patients with low IL-22 concentrations who were treated with TNF-i and those with high IL-22 concentrations who were treated with IL-17-i. Table 2 shows the baseline characteristics of the strategic treatment group (17 cases) and the mismatched treatment group (17 cases). No statistically significant differences were observed between the two groups in baseline characteristics at the initiation of bDMARD therapy. There were no statistically significant differences between the groups with respect to the concomitant administration of MTX. There were also no significant differences in the percentages of patients who received TNF-i or IL-17-i (Table 2). Table 3 also shows the treatment responses over 1 year. After initiation of bDMARD therapy, tender joint counts, swollen joint counts, C-reactive protein (CRP), DAPSA, and PASI were significantly improved in both groups. When the treatment responses over 1 year were compared between the two groups, the rate of achieving Minimal Disease Activity at month 6, DAPSA-REM, and Minimal Disease Activity at month 12 was significantly higher in the strategic treatment group. There were no statistically significant differences in the rates of achieving PASI75 or PASI90 at month 6 or 12 between the two groups (Table 3).

**Table 2:** Baseline characteristics of the strategic treatment group and mismatched treatment group

Variables	Strategic treatment (*n*=17)	Mismatched (***n***=17)	***p*** value
**Age (years)**	53.2±16.0	58.4±10.8	0.5016
**Male,** ***n*** **(%)**	12 (70.5)	6 (35.3)	0.0084
**Disease duration (months)**			
**PSO**	108 (42, 288)	216 (10, 258)	0.9176
**PsA**	42 (13.5, 116.5)	43 (16.5, 144)	0.7960
**Peripheral arthritis**	17 (100)	17(100)	1.0000
**Spinal involvement**	4 (23.5)	6 (35.2)	0.7080
**History of past bDMARDs**	1st 12 (70.5)	1st 12 (70.5)	1.0000
**Concomitant MTX use**	12 (70.6)	10 (58.8)	0.7270
**bDMARDS initiated**			
**IL-17-i/TNF-i**	6 (35.2)/11 (64.7)	8 (47.0)/9 (52.9)	0.4905
**Disease activity**			
**TJC**	7 (5, 8.5)	8 (3.5, 14)	0.4164
**SJC**	6 (4, 12.5)	5 (2.5, 7)	0.1453
**CRP (mg/dl)**	0.99 (0.18, 2.14)	0.54 (0.075, 1.71)	0.2347
**DAPSA**	24.1 (19.2, 33.7)	31.2 (20.9, 39.8)	0.1848
**PASI**	3.1 (0.5, 5.75)	1.6 (0.4, 6.3)	0.7823

Data are expressed as mean ± standard deviation, median (interquartile range [IQR]), or number (%)

*TNF-i* TNF inhibitors, *IL-17-i* IL-17 inhibitors, *TJC* tender joint counts 68, *SJC* swollen joint counts 66, *DAPSA* Disease Activity in Psoriatic Arthritis, *PASI* Psoriasis Area and Severity Index

*p<0.05, by Mann-Whitney *U* test or chi-square test

**Table 3:** Comparison of 1 year treatment response between the strategic treatment group and unmatched treatment group

Variables	Strategic treatment (***n***=17)	mismatched (***n***=17)	***p***-value
**Month 6**			
**⊿TJC (M6-BL)**	− 6 (− 8, − 4.5)	− 5 (− 11, − 2.5)	0.9172
**⊿SJC (M6-BL)**	− 4 (− 7, − 2)	− 4 (− 10.5, − 2)	0.8086
**⊿DAPSA (M6-BL)**	− 18.6 (− 27.3, − 13.7)	− 18.4 (− 32.4, − 13.3)	0.9314
**% decrease, SDAI**	− 77.7 (− 89.9, − 71.4)	− 74.0 (− 82.3, − 53.2	0.1432
**Proportion of REM,** ***n*** **(%)**	7 (41.1)	3 (17.6)	0.2587
**Proportion of REM/LDA,** ***n*** **(%)**	17 (100)	14 (82.3)	0.1136
**Minimal Disease Activity**	12 (70.6)	4 (25.3)	0.0075*
**% improvement, PASI**	86.0 (53.1, 100)	100 (17.6, 100)	0.9809
**PASI90,** ***n*** **(%)**	7/15 (46.7)	9/14 (64.2)	0.9469
**PASI75,** ***n*** **(%)**	7/15 (46.7)	9/14 (64.2)	0.9469
**Month 12**			
**⊿TJC (M12-BL)**	− 6 (− 8.5, − 3)	− 5 (− 11.5, − 2)	0.9175
**⊿SJC (M12-BL)**	− 4 (− 6, − 1.5)	− 5 (− 11.5, − 2)	0.4365
**⊿DAPSA**	− 18.4 (− 27.3, − 12.9)	− 19.45 (− 33.6, − 10.5)	0.8228
**% decrease, DAPSA (M12)**	− 87.4 (− 97.1, − 75.4)	− 72.0 (− 89.2, − 44.7)	0.0605
**Proportion of REM,** ***n*** **(%)**	10 (58.8)	4 (25.3)	*0.0399*
**Proportion of REM/LDA,** ***n*** **(%)**	15 (88.2)	13 (76.4)	0.3281
**Minimal Disease Activity**	14 (82.3)	7 (41.2)	*0.0162*
**% improvement, PASI**	92.7 (51.7, 100)	100 (72.7, 100)	0.3794
**PASI90,** ***n*** **(%)**	9/15 (60)	10/14 (71.4)	0.8502
**PASI75,** ***n*** **(%)**	9/15 (60)	11/14 (78.5)	0.9320

Data are expressed as median (interquartile range [IQR] ) or number (%)

*TNF-i* TNF inhibitors, *IL-17-i* IL-17 inhibitors, *TJC* tender joint counts 68, *SJC* swollen joint counts 66, *DAPSA* Disease Activity in Psoriatic Arthritis, *LDA* low disease activity, *REM* remission, *PASI* Psoriasis Area and Severity Index

*p<0.05, by Mann-Whitney *U* test or chi-square test

## Discussion

This report is the first to demonstrate the possibility of optimizing the selection between TNF-i and IL-17-i based on serum IL-22 concentrations.

PsA is a disease with very high heterogeneity, and its clinical symptoms vary among patients. Thus, we focused on serum cytokine concentrations as biomarkers that could contribute to simple and proper drug selection. In addition, we previously reported that drug selection based on phenotyping of peripheral blood lymphocytes leads to more frequent achievement of low disease activity as assessed by the SDAI [9]. In the present study, we aimed to develop treatment strategies that would lead to the achievement of DAPSA-REM, PASI90, or Minimal Disease Activity and more effective control of PsA.

Since treatment was selected based on shared decision-making between the attending physicians and the patients in this non-randomized study, there were statistically significant differences in the prevalence of comorbid diabetes mellitus and serum IL-17A concentrations measured after the end of the observation period in cohort 1 (Supplementary Table S1). However, we continued the analyses described below, because we did not intend to directly compare the efficacy of TNF-i and IL-17-i. Instead, we aimed to identify cytokines predictive of responses to each of the TNF-i and IL-17-i therapies and to develop new treatment strategies based on the identified cytokines.

Univariate logistic analyses identified serum IL-22 concentrations as a factor contributing to the achievement of DAPSA-REM in IL-17-i therapy (Supplementary Table S3).

Patients in cohort 1 were stratified based on serum IL-22 concentrations, and the efficacy of IL-17-i and TNF-i therapies was evaluated. The results showed that significantly more patients with low IL-22 concentrations who were treated with IL-17-i achieved DAPSA-REM and Minimal Disease Activity (Table 1). Meanwhile, a comparison of the baseline characteristics showed significant differences in the concomitant use of MTX (Supplementary Table S4). We considered the possibility that the difference in the proportions of patients with spinal involvement may have affected the administration of MTX. However, there were no significant differences in the proportions of patients with spinal involvement. We also considered the possibility that the concentrations of IL-22 differed between patients with spinal involvement (both peripheral arthritis and spinal involvement) and those without (peripheral arthritis alone), but there was no significant difference in the concentrations of IL-22 between the two groups (Supplementary Fig. S1(A)). Although we considered that low IL-22 concentrations may reflect the low inflammatory state due to MTX, no statistically significant differences were observed in the baseline values of CRP, or serum IL-22 concentrations between the 29 patients with concomitant use of MTX and the 18 patients without in cohort 1 (Supplementary Fig. S1(B)). Similarly, there was no significant difference in serum IL-17 concentrations between these two groups with and without concomitant use of MTX. Since serum concentrations of IL-17 and IL-22 did not correlate with CRP (Supplementary Fig. S1(C)), the levels of CRP and the concomitant use of MTX seemed unlikely to reflect the serum concentrations of IL-17 and IL-22.

Thus, we focused on IL-22 concentrations as a simple biomarker for the proper selection of TNF-i and IL-17-i to develop treatment strategies.

Moreover, no factors contributing to the achievement of PASI90 were identified for either TNF-i or IL-17-i therapy (Supplementary Table S4), and no differences were observed in efficacy in terms of PASI during treatment (Tables 1 and 3). A low PASI score at baseline may have affected the results.

In cohort 2 (validation cohort), we compared treatment responses over 1 year between the strategic and mismatched treatment groups. The results showed that the rate of achieving DAPSA-REM and Minimal Disease Activity at 1 year was significantly higher in the strategic treatment group (Table 3). As we hypothesized, it was suggested that patients with PsA may include two types of patients. In the first type of patients, Th17 cells and ILCs, which are involved in IL-17 production, are mainly involved in pathogenesis, which is consistent with the conventional assumption. In the second type of patients, Th22 cells, which are involved in IL-22 production, are also involved in the pathogenesis of this disease. Future studies on the correlation between patients with high IL-22 concentrations and Th22 cell subsets in peripheral blood and on changes in serum concentrations of IL-22 and Th22 cells due to treatment are expected to advance the elucidation of the pathology with a focus on the Th22-IL-22 correlation.

This study excluded patients using IL-12/23 (p40) inhibitors, because no clinical studies have demonstrated that IL-12/23 (p40) inhibitors are comparable to TNF-i or IL-17-i. This study focused on the proper selection of only TNF-i and IL-17-i. Moreover, patients using IL-23 (p19) inhibitors, which were approved in Japan only shortly before this study, were also excluded due to an insufficient number of patients. Based on our hypothesis that there are patients whose main pathogenic factors are Th17 cells and ILCs, which are involved in IL-17 production, and patients in whom Th22 cells involved in IL-22 production are also involved in the pathogenesis, IL-23 (p19) inhibitors may be more effective in the former. In addition, the use of JAK-i has recently expanded to include the treatment of PsA [11, 12]. Since JAK-i is expected to inhibit the differentiation of Th17 cells as well as Th22 cells, it appears to be a potential drug that is highly effective for a wide spectrum of PsA.

## Conclusions

The results of this pilot study suggest that IL-22 may be a biomarker of response to TNF-i and IL-17-i in patients with PsA. Further large-scale studies using independent, prospectively collected datasets are required to claim that IL-22 is indeed a biomarker of treatment response.

In this study, DAPSA was used to assess disease activity because all patients had peripheral arthritis. However, it is possible that the assessment method used in this study was insufficient because we included patients with and without spinal involvement. It is also possible that this heterogeneity may have affected treatment selection. Given the heterogeneity of PsA patients, the difficulties in measuring response, and the potential differences in treatments, even within the same class, larger cohorts are required.

## Supplementary Information

**Additional file 1: Supplementary Figure S1.** Differences between subgroups in cohort 1. (A) Comparison of IL-22 concentrations between patients with spinal involvement (+) (n =14) and patients without spinal involvement (-) (n= 33). (B) Comparison of IL-17A, IL-22, and CRP concentrations in patients treated with or without MTX. Methotrexate (MTX)+: 29 patients, MTX-: 18 patients. Data are presented as median (IQR). *p <0.05, Mann-Whitney U test. (C). Correlation between CRP and serum concentrations of IL-17A and IL-22 in cohort 1. *p <0.05, by a simple linear regression test.

**Additional file 2: Supplementary Table S1.** Baseline characteristics of the patients in cohort 1.

**Additional file 3: Supplementary Table S2.** Comparison of 1 year treatment response between TNF-i and IL-17-i in cohort 1.

**Additional file 4: Supplementary Table S3.** Baseline predictive serum cytokine of MDA/DAPSA-remission/PASI90 by IL-17 or TNF-i analyzed by univariate analysis.

**Additional file 5: Supplementary Table S4.** Comparison of baseline characteristics between IL-22 high and low group.

## Data Availability

Not applicable

## Abbreviations

TNF-i: Tumor necrosis factor inhibitors interleukin
IL-17-i: Interleukin-17 inhibitors
PsA: Psoriatic arthritis
PS: Psoriasis
IFN-γ: Interferon γ
IL: Interleukin
JAK-i: Janus kinase inhibitors
PDE4-i: Phosphodiesterase 4 inhibitors
DAPSA: Disease Activity in Psoriatic Arthritis
PASI: Psoriasis area and severity index
REM: Remission
HC: Healthy control
CCR: Chemokine receptor
