# Supplementary figures and images for: Impact of serum interleukin-22 as a biomarker for the differential use of molecular targeted drugs in psoriatic arthritis: a retrospective study
Source: Arthritis Res Ther. 2022 Apr 15;24:86. doi: 10.1186/s13075-022-02771-4 (PMC9011943; doi:10.1186/s13075-022-02771-4)

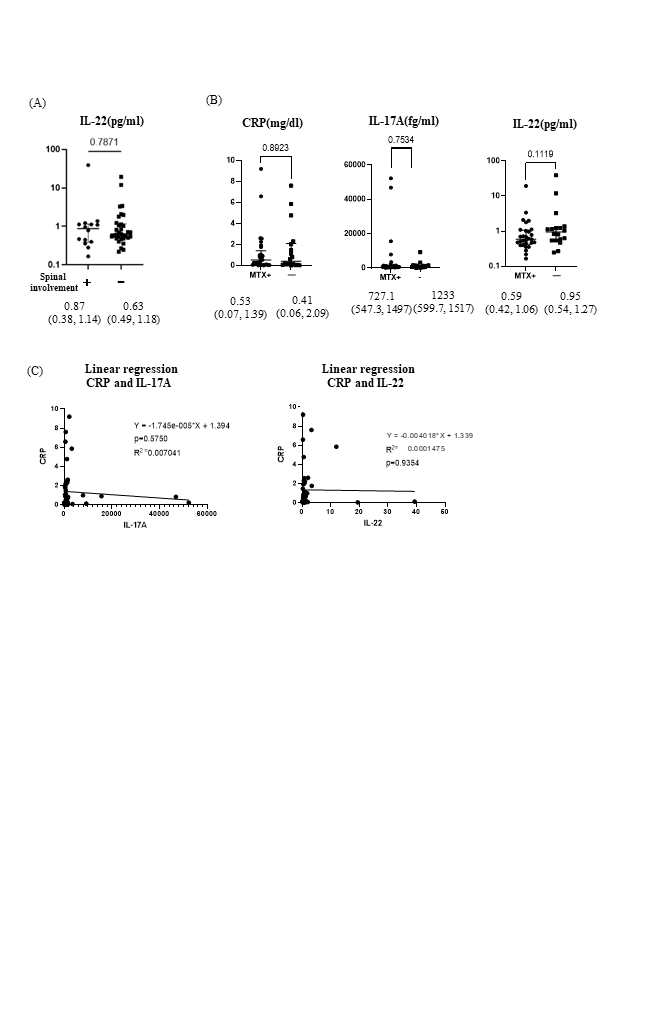

Supplement: Supplementary file 1 — Additional file 1: Supplementary Figure S1. Differences between subgroups in cohort 1. (A) Comparison of IL-22 concentrations between patients with spinal involvement (+) (n =14) and patients without spinal involvement (-) (n= 33). (B) Comparison of IL-17A, IL-22, and CRP concentrations in patients treated with or without MTX. Methotrexate (MTX)+: 29 patients, MTX-: 18 patients. Data are presented as median (IQR). *p <0.05, Mann-Whitney U test. (C). Correlation between CRP and serum concentrations of IL-17A and IL-22 in cohort 1. *p <0.05, by a simple linear regression test. [file 13075_2022_2771_MOESM1_ESM.tif]
