# Supplementary material for: Impact of serum interleukin-22 as a biomarker for the differential use of molecular targeted drugs in psoriatic arthritis: a retrospective study
Source: Arthritis Res Ther. 2022 Apr 15;24:86. doi: 10.1186/s13075-022-02771-4 (PMC9011943; doi:10.1186/s13075-022-02771-4)
Supplement: Supplementary file 2 — Additional file 2: Supplementary Table S1. Baseline characteristics of the patients in cohort 1. [file 13075_2022_2771_MOESM2_ESM.docx]

**Supplementary Table S1. Baseline characteristics of the patients in cohort 1**

| **Variables** | **All (n=47)** | **TNF-i (n=24)** | **IL-17-i (n=23)** | **p-value** |
| --- | --- | --- | --- | --- |
| **Age** | 53.3±15.5 | 57.2±3.09 | 49.3±3.16 | 0.0790 |
| **Male, n (%)** | 24(51.0) | 10(41.6) | 14(60.8) | 0.2460 |
| **BMI (Kg/m^2^)** | 24.4±4.31 | 23.4±0.86 | 25.5±0.88 | 0.0847 |
| **Disease duration** |  |  |  |  |
| **PSO** | 174.6±164.6 | 178.5±33.9 | 170.6±34.6 | 0.9915 |
| **PsA(months)** | 121.7±17.7 | 98.8±24.9 | 70.6±25.4 | 0.1939 |
| **Onset (skin ahead/ arthritis ahead/simultaneous), n (%)** | 31(69.5)/9(19.1)/7(14.8) | 14(58.3)/6(25.0)/4(16.7) | 17(73.9)/2(13.0)/3(13.0) | 0.4935 |
| **Preceding duration**  **(Skin-arthritis) (months)** | -89.6(-180, 0) | -21.5(-212.2, 28.5) | -24(-165, 0) | 0.6164 |
| **Clinical symptoms** |  |  |  |  |
| **Peripheral arthritis** | 47(100) | 24(100) | 23(100) | 1.0000 |
| **Spinal involvement** | 14(29.7) | 7(29.1) | 7(30.4) | 1.0000 |
| **Eye, n (%)** | 1(2.1) | 1(4.17) | 0(0) | 1.0000 |
| **Valvular disease/IBD, n (%)** | 0(0) | 0(0) | 0(0) | N.A. |
| **DM** | 13(27.6) | 10(41.6) | 3(13.0) | ***0.0490** |
| **DL** | 8(17.0) | 6(25.0) | 2(8.70) | 0.2448 |
| **TJC** | 6(2, 9) | 5.5(2.25, 8.75) | 6(2, 9) | 0.6926 |
| **SJC** | 4(1, 6) | 5(1, 10.5) | 3(1, 6) | 0.2139 |
| **CRP (mg/dl)** | 0.53(0.07, 1.75) | 0.41(0.04, 1.96) | 0.53(0.1, 1.46) | 0.7414 |
| **DAPSA** | 21.9(13.3, 32.3) | 22.1(14.6, 31.8) | 21.1(12.8, 32.6) | 0.8149 |
| **PASI** | 2(0.6, 6.4) | 3.2(0.825, 5.77) | 1.6(0.6, 7.2) | 0.8898 |
| **IFN-γ (pg/ml)** | 8.53(5.69, 11.6) | 8.67(6.20, 12.5) | 7.98(4.50, 10.9) | 0.6170 |
| **IL-6 (pg/ml)** | 3.43(0.96, 7.97) | 4.08(1.09, 8.45) | 1.71(0.89, 5.71) | 0.1906 |
| **TNF-α (pg/ml)** | 2.11(1.54, 3.94) | 2.10(1.43, 4.19) | 2.11(1.57, 3.94) | 0.8815 |
| **IL-17A (fg/ml)** | 782.0(548.5, 1501.3) | 676.6(527.7, 1237.9) | 1361.9(684.3, 2179.7) | ***0.0083** |
| **IL-21 (pg/ml)** | 1.47(0, 3.27) | 0.50(0, 2.63) | 1.81(0, 5.89) | 0.1713 |
| **IL-22 (pg/ml)** | 0.68(0.46, 1.14) | 0.65(0.45, 1.62) | 0.68(0.50, 1.14) | 0.8732 |
| **IL-23 (pg/ml)** | 0.54(0, 1.37) | 0.38(0, 1.36) | 0.69(0, 1.42) | 0.6548 |

Data are expressed as mean ± standard deviation, median (interquartile range [IQR]), or number (%).

TNF-i: TNF inhibitors, IL-17-i: IL-17-inhibitors, DM: diabetes mellitus, DL: dyslipidemia, TJC: tender joint counts (66), SJC: swollen joint counts (68), DAPSA: disease activity in psoriatic arthritis, PASI: psoriasis area and severity index, N.A.: not applicable, *p<0.05, TNF-i vs. IL-17-i), by Mann–Whitney U test or chi-square test
