# Supplementary material for: Impact of serum interleukin-22 as a biomarker for the differential use of molecular targeted drugs in psoriatic arthritis: a retrospective study
Source: Arthritis Res Ther. 2022 Apr 15;24:86. doi: 10.1186/s13075-022-02771-4 (PMC9011943; doi:10.1186/s13075-022-02771-4)
Supplement: Supplementary file 3 — Additional file 3: Supplementary Table S2. Comparison of 1 year treatment response between TNF-i and IL-17-i in cohort 1. [file 13075_2022_2771_MOESM3_ESM.docx]

**Supplementary Table S2. Comparison of 1 year treatment response between TNF-i and IL-17-i in cohort 1**

| **Variables** | **TNF-i (n=24)** | **IL-17-i (n=23)** | **p-value** |
| --- | --- | --- | --- |
| **Month 6** |  |  |  |
| **⊿TJC (M6-BL)** | -5(-6.75, -2) | -5(-8, -2) | 0.9915 |
| **⊿SJC (M6-BL)** | -3(-9, -1) | -2(-5, -1) | 0.3565 |
| **⊿DAPSA (M6-BL)** | -14.1(-26.1, -8.51) | -15.7(-21.8, -7.3) | 0.9961 |
| **% Decrease-SDAI** | -80.2(-87.2, -59.3) | -76.7(-94.5, -59.6) | 0.8648 |
| **Proportion of REM, n(%)** | 12(50.0) | 8(40.0) | 0.3801 |
| **Proportion of REM/LDA, n(%)** | 22(91.6) | 22(95.6) | 1.0000 |
| **Minimal disease activity, n (%)** | 14(58.3) | 14(60.8) | 1.0000 |
| **% Improvement-PASI** | 93.0(44.7, 100) | 100(33.3, 100) | 0.7116 |
| **PASI90, n (%)** | 11/22(50.0) | 11/18(61.1) | 0.5371 |
| **PASI75, n (%)** | 14/22(63.6) | 12/18(66.6) | 1.0000 |
|  |  |  |  |
| **Month 12** |  |  |  |
| **⊿TJC (M12-BL)** | -4(-7.75, -2) | -4(-9, -2) | 0.7420 |
| **⊿SJC (M12-BL)** | -3.5(-6, -0.25) | -3(-5, -1) | 0.6840 |
| **⊿DAPSA** | -14.8(-25.7, -5.2) | -14.9(-26.7, -7.2) | 0.7017 |
| **% Decrease-DAPSA (M12)** | -82.2(-94.8, -36.4) | -77.2(-95.0, -62.5) | 0.8648 |
| **Proportion of REM, n(%)** | 13(54.2) | 11(47.8) | 0.7732 |
| **Proportion of REM/LDA, n(%)** | 19(79.2) | 22(95.6) | 0.1882 |
| **Minimal disease activity, n (%)** | 17(70.8) | 16(69.6) | 1.0000 |
| **% Improvement-PASI** | 93.3(52.7, 100) | 100(87.5, 100) | 0.4197 |
| **PASI90, n (%)** | 12/22(54.5) | 14/18(77.8) | 0.1860 |
| **PASI75, n(%)** | 15/22(68.2) | 14/18(77.8) | 0.7235 |

Data are expressed as median (interquartile range [IQR]) or number (%).

TNF-i: TNF inhibitors; IL-17-i: IL-17-inhibitors; TJC: tender joint counts (66), SJC: swollen joint counts (68), DAPSA: disease activity in psoriatic arthritis; LDA: low disease activity; REM: remission; PASI: psoriasis area and severity index, *p<0.05, by Mann–Whitney U test or chi-square test.
