# Supplementary material for: Impact of serum interleukin-22 as a biomarker for the differential use of molecular targeted drugs in psoriatic arthritis: a retrospective study
Source: Arthritis Res Ther. 2022 Apr 15;24:86. doi: 10.1186/s13075-022-02771-4 (PMC9011943; doi:10.1186/s13075-022-02771-4)
Supplement: Supplementary file 4 — Additional file 4: Supplementary Table S3. Baseline predictive serum cytokine of MDA/DAPSA-remission/PASI90 by IL-17 or TNF-i analyzed by univariate analysis. [file 13075_2022_2771_MOESM4_ESM.docx]

**Supplementary Table S3. Baseline predictive serum cytokine of MDA/DAPSA-remission/PASI90 by IL-17 or TNF-i analyzed by univariate analysis**

| **IL-17-inhibitors** | **Wald** | **p-value** | **OR (95 %CI)** |
| --- | --- | --- | --- |
| **【Minimal disease activity】** |  |  |  |
| **IFN-γ** | 0.39 | 0.52 | 1.03(0.93, 1.14) |
| **IL-6** | 0.13 | 0.71 | 0.97(0.87, 1.09) |
| **TNF-α** | 0.06 | 0.79 | 1.02(0.84, 1.24 |
| **IL-17A** | 0.32 | 0.56 | 0.99(0.99, 1.00) |
| **IL-21** | 0.40 | 0.52 | 0.95(0.84, 1.09) |
| **IL-22** | 0.03 | 0.84 | 0.95(0.62, 1.46) |
| **IL-23** | 1.13 | 0.28 | 1.24(0.83, 1.85) |
| **【DAPSA-REM】** |  |  |  |
| **IFN-γ** | 2.56 | 0.11 | 1.19(0.96, 1.48) |
| **IL-6** | 2.59 | 0.10 | 1.29(0.94, 1.78) |
| **TNF-α** | 0.77 | 0.37 | 1.10(0.88, 1.36) |
| **IL-17A** | 1.13 | 0.28 | 0.99(0.99, 1.00) |
| **IL-21** | 0.39 | 0.53 | 0.99(0.96, 1.01) |
| **IL-22** | 4.71 | **0.02*** | 36.3(1.41, 929.2)) |
| **IL-23** | 0.15 | 0.69 | 1.08(0.73, 1.60)) |
| **【PASI 90】** |  |  |  |
| **IFN-γ** | 0.02 | 0.87 | 1.00(0.87-1.12) |
| **IL-6** | 1.25 | 0.26 | 0.45(0.11-1.80) |
| **TNF-α** | 0.33 | 0.56 | 0.91(0.54-1.16) |
| **IL-17A** | 0.67 | 0.41 | 1.00(0.99, 1.00) |
| **IL-21** | 0.24 | 0.61 | 0.98(0.87, 1.02) |
| **IL-22** | 0.09 | 0.75 | 0.91(0.50, 1.63) |
| **IL-23** | 1.10 | 0.29 | 1.24(0.82, 1.87) |
| **TNF-inhibitors** |  |  |  |
| **【Minimal disease activity】** |  |  |  |
| **IFN-γ** | 3.14 | 0.17 | 1.27(0.97, 1.66) |
| **IL-6** | 0.60 | 0.43 | 1.03(0.94, 1.13) |
| **TNF-α** | 1.32 | 0.25 | 1.10(0.93, 1.30) |
| **IL-17A** | 0.10 | 0.74 | 1.00(0.99, 1.00) |
| **IL-21** | 0.11 | 0.73 | 1.05(0.79, 1.38) |
| **IL-22** | 0.54 | 0.46 | 1.34(0.61, 2.93) |
| **IL-23** | 0.62 | 0.42 | 0.75(0.37, 1.51) |
| **【DAPSA-REM】** |  |  |  |
| **IFN-γ** | 0.20 | 0.64 | 1.02(0.99-1.24) |
| **IL-6** | 0.81 | 0.36 | 0.93(0.80-1.08) |
| **TNF-α** | 0.32 | 0.57 | 1.04(0.90-1.20) |
| **IL-17A** | 0.41 | 0.51 | 1.00(0.99-1.00) |
| **IL-21** | 0.08 | 0.76 | 0.95(0.68-1.32) |
| **IL-22** | 1.05 | 0.30 | 1.05(0.95-1.17) |
| **IL-23** | 0.00 | 0.96 | 1.01(0.53-1.93) |
| **【PASI-90】** |  |  |  |
| **IFN-γ** | 0.13 | 0.71 | 1.01(0.99-1.24) |
| **IL-6** | 0.68 | 0.40 | 1.04(0.95-1.19) |
| **TNF-α** | 0.65 | 0.41 | 1.06(0.91-1.28) |
| **IL-17A** | 0.80 | 0.36 | 0.99(0.99-1.00) |
| **IL-21** | 0.25 | 0.61 | 1.07(0.80-1.50) |
| **IL-22** | 0.03 | 0.85 | 0.99(0.86-1.10) |
| **IL-23** | 1.36 | 0.24 | 0.58(0.18-1.20) |

TNF-i, TNF inhibitors; IL-17-i, IL-17-inhibitors; MDA, minimal disease activity; DAPSA, disease activity in psoriatic arthritis; REM, remission; PASI, psoriasis area and severity index.
