# Supplementary material for: Impact of serum interleukin-22 as a biomarker for the differential use of molecular targeted drugs in psoriatic arthritis: a retrospective study
Source: Arthritis Res Ther. 2022 Apr 15;24:86. doi: 10.1186/s13075-022-02771-4 (PMC9011943; doi:10.1186/s13075-022-02771-4)
Supplement: Supplementary file 5 — Additional file 5: Supplementary Table S4. Comparison of baseline characteristics between IL-22 high and low group. [file 13075_2022_2771_MOESM5_ESM.docx]

**Supplementary Table S4. Comparison of baseline characteristics between IL-22 high and low group**

| **Variables** | **IL-22 low** | **IL-22 high** | **p-value** |
| --- | --- | --- | --- |
| **TNF-i treated group** | **n=12** | **n=12** |  |
| **Age (years)** | 57.2±12.0 | 57.3±17.2 | 0.4526 |
| **Male, n (%)** | 4(33.3) | 6(50) | 0.6802 |
| **Disease Duration (months)** |  |  |  |
| **PSO (month)** | 228(7.75, 291.7) | 115.5(27, 318) | 0.8852 |
| **PsA (month)** | 46(17.7, 124.5) | 48(12.7, 217.5) | 0.9080 |
| **Peripheral arthritis** | 12(100) | 12(100) | 1.0000 |
| **Spinal involvement** | 3(25.0) | 4(33.3) | 1.0000 |
| **History of past bDMARDs** | 1^st^ 10(83.3) | 1^st^ 9 (75.0) | 1.0000 |
| **Concomitant MTX use** | 6(50) | 5(41.6) | 1.0000 |
| **TJC** | 5(2, 14) | 5.5(3.5, 7) | 0.8391 |
| **SJC** | 6(1.5, 14.5) | 4(1, 6) | 0.1624 |
| **CRP (mg/dl)** | 0.14(0.02, 0.74) | 1.01(0.12, 2.19) | 0.0884 |
| **DAPSA** | 23.9(9.0, 42.0) | 21.2(16.2, 26.0) | 0.6033 |
| **PASI** | 1.8(0.65, 7.8) | 3.8(1.07, 5.77) | 0.4527 |
| **IL-17 treated group** | **n=10** | **n= 13** |  |
| **Age (years)** | 44.1±14.0 | 53.2±16.4 | 0.1448 |
| **Male, n (%)** | 8(80) | 6(46.2) | 0.1968 |
| **Disease Duration (months)** |  |  |  |
| **PSO (month)** | 209(41, 345) | 72(33, 171) | 0.2507 |
| **PsA (month)** | 34(17.2, 49.2) | 32(5.5, 96) | 0.9258 |
| **Peripheral arthritis** | 10(100) | 13(100) | 1.0000 |
| **Spinal involvement** | 2(20.0) | 5(38.4) | 0.4050 |
| **History of past bDMARDs** | 1^st:^ 8(80) | 1^st^ 6 (46.1) | 0.1968 |
| **Concomitant MTX use** | 1(10) | 8(61.5) | **0.0288*** |
| **TJC** | 6(3.75, 8.5) | 3(1, 9.5) | 0.4357 |
| **SJC** | 3(2, 8.25) | 3(0, 5.5) | 0.3640 |
| **CRP (mg/dl)** | 0.44(0.18, 1.57) | 0.62(0.06, 1.71) | 0.5350 |
| **DAPSA** | 21.5(12.0, 36.6) | 19.7(13.2, 32.4) | 0.9013 |
| **PASI** | 1.15(0.55, 6) | 2(0.3, 13.6) | 0.4187 |

Data are expressed as mean ± standard deviation, median (interquartile range [IQR]) or number (%).

TNF-i: TNF inhibitors; IL-17-i: IL-17-inhibitors; TJC: tender joint counts 68; SJC: swollen joint counts 66; DAPSA, disease activity in psoriatic arthritis; PASI: psoriasis area and severity index *p<0.05, by Mann–Whitney U test or chi-square test
